# The associations between Toll-like receptor 4 gene polymorphisms and hepatitis C virus infection: a systematic review and meta-analysis

**DOI:** 10.1042/BSR20182470

**Published:** 2019-02-26

**Authors:** Narttaya Chaiwiang, Teera Poyomtip

**Affiliations:** Faculty of Optometry, Ramkhamhaeng University, Bangkok, Thailand

**Keywords:** Hepatitis C virus (HCV), Meta-analysis, Polymorphisms, Toll-like receptor 4 (TLR4)

## Abstract

**Background and objective:** The hepatitis C virus (HCV) is able to cause a life-threatening disease relating to lethal hepatocellular carcinoma. Previous, Toll-like receptor polymorphisms were proposed as promising biomarker for HCV-related hepatocellular carcinoma and disease progression. This study aimed to summarize the association of TLR4 polymorphisms and HCV infection through meta-analysis.

**Methods:** We applied a systematic review and meta-analysis performed by using PubMed, EMBASE and Web of Science searches. The Modified Newcastle-Ottawa scale was used for quality assessment. The odd-ratio (OR) and 95% confidence interval (CI) were calculated to assess the association. *In silico* analysis was applied for proposing the function as microRNA (miRNA) of non-coding polymorphism. Finally, the miRNA target was predicted and annotated to suggest the possible relationship between polymorphism and HCV infection.

**Results:** Our meta-analysis incorporated seven studies involving rs4986791, rs4986790 and rs2149356. No association exists between rs4986791 and HCV infection. However, the heterozygous model (AG vs GG) of rs4986790 significantly associates with HCV infection (OR = 0.33, 95% CI = 0.21–0.49, *P*<0.0001). Moreover, the rs2149356 TG genotype also associates with HCV infection in the over-dominant model (TG vs TT+TG: OR = 0.54, 95% CI = 0.40–0.75). *In silico* analysis of rs2149356G allele showed that this mutation is siRNA, which targets the set of genes, especially in the autophagy pathway.

**Conclusion:** We demonstrated that rs4986790 and rs2149356 are associated with HCV infection.

## Introduction

Hepatitis C virus (HCV), which is composed of 6 genotypes and 90 subtypes, causes a disease that is life-threatening to mankind since it is associated with highly lethal hepatocellular carcinoma [1,2]. The virus infects approximately 180 million people worldwide and has led to a global public health problem due to the fact that infection with HCV mainly results in asymptomatic infection and sub-clinical manifestation [3]. Moreover, HCV patients may develop non-specific symptoms such as fatigue, weakness, appetite loss and muscle pain. Therefore, HCV infection is a silent pandemic [4]. Importantly, the vast majority of patients infected with HCV are inapparent cases that cause viral circulation in the population. Acute infection is capable of progressing into a chronic disease, cirrhosis and HCC, resulting in an estimated 700,000 deaths per year [5,6].

Innate immunity is a crucial response to pathogens, including HCV infection. Among such innate molecules, Toll-like receptors (TLRs) interact with pathogen-associated molecular patterns such as lipopolysaccharides (LPS), peptidoglycan and viral RNA and DNA. Nowadays, approximately 10 TLRs are identifiable in humans and located in different compartments of the cells. These receptors respond to various pathogens, including both extracellular and intracellular microorganisms. In viral infections, TLR activation promotes antiviral response in an interferon-dependent manner [7]. Particularly, TLR4 is the first discovered and has been a well-known topic of study for a long time [8]. Evidence has previously shown that TLR4, located on chromosomes 9q32-33, is able to recognize the lipoprotein (NS1) of dengue virus and NS5A of HCV [9,10].

In recent decades, personal medicine has emerged as a novel therapy based on the best response and highest safety of patients. To achieve this regard, gene association studies are required for personalized information. Interestingly, TLR4 single nucleotide polymorphisms (SNPs) have been reported as a risk factor in several infectious diseases such as Malaria, Hepatitis A virus and Hepatitis E virus [11–13]. At present, increasing evidence suggests that SNPs in TLR4 are associated with several steps of HCV outcome. Li et al., [14] showed the multiple variables in TLR4 are a risk factor of liver fibrosis in Caucasians with chronic hepatitis C infection. In addition, TLR4 rs4986790 (Asp299Gly) and rs4986791 (Thr399Ile) polymorphisms are associated with viral loading and delayed successive antiviral therapy as discuss by Peric et al. [15]. Consequently, Sghaier et al. suggested that A allele and AA genotype of TLR4 rs4986790 play a role as protective factors in chronic HCV infection and tend to occur in pegylated IFN-α and Ribavirin responder subjects at a higher rate than non-responder subjects [16]. The number of studies showing the relation between TLR4 SNPs and HCV susceptibility is under-reported. There are two studies indicating that both rs4986790 and rs4986791 are associated with HCV infection in Saudi Arabia and Pakistan [17,18]. However, these results remain controversial as some other studies could not detect any association [19–21]. Moreover, other TLR4 SNPs might play other roles in HCV infection. Previous evidence has suggested that rs2149356 significantly reduced the risk of HCC and delayed interferon therapy. However, this position lacks association evidence which is related to HCV infection [22,23].

To increase the validity of TLR4 rs4986790 and rs4986791 association with HCV infection, we conducted a systematic review and meta-analysis. Moreover, our study assesses whether there is a relation between TLR4 rs2149356 and HCV. We demonstrated, for the first time by using *in silico* analysis, that TLR4 rs2149356G is a part of miRNA sequences, which control groups of genes. Our work will provide one piece of the puzzle to filling the gap in knowledge concerning HCV infection.

## Materials and methods

### Literature search

We performed a systematic review based on electronic literature databases in PubMed, Excerpta Medical Database (EMBASE) and Web of Science. The search was carried out and updated until 26 December 2018. The following keywords were used: ((HCV OR Hepatitis C) AND (variants OR Genetic Polymorphisms OR Genetic Polymorphism OR genotyping OR SNP OR SNPs OR Single Nucleotide Polymorphism OR Single Nucleotide Polymorphisms OR Polymorphisms OR Polymorphism OR Nucleotide Polymorphism) AND (TLR-4 OR TLR4 OR Receptor, TLR4 OR TLR4 Receptor OR Toll 4 Receptor OR Toll-4 Receptor OR Toll Like Receptor 4)). Two principal investigators independently searched the aforementioned databases and subsequently screened the title and abstract. To decide on the inclusion and exclusion criteria for disagreement, the authors held discussions to resolve issues for final consensus. Related review articles were screened to identify additional data.

### Inclusion and exclusion criteria

Selected publications included in our study were: (1) case-control or cohort study on the association of TLR4 gene polymorphisms and HCV infection; (2) Odd-ratio (OR) or relative risk with 95% confidence interval (CI); (3) human study. The exclusion criteria were: (1) Abstract, case-report review, systematic review and other non-original studies; (2) liver transplantation; (3) non-relevant study; (4) co-infection with other microorganisms; (5) lack of data to calculate genetic models; (6) lack of author information to access the full paper in other languages.

### Quality assessment

Two independent authors evaluated the research quality according to the modified Newcastle-Ottawa scale (NOS) for genetic association study [24]. The NOS criterion is divided into three categories: (1) subject selection; (2) the comparability of subject; (3) exposure. The total score is nine, with zero to four classified as a low-quality study, five to six classified as a moderate-quality study and seven to nine classified as a high-quality study. The moderate-quality and high quality studies were included into meta-analysis. Any disagreement was discussed for resolution before any final decision.

### Data extraction

The data were extracted by using a data extraction form. The necessary information from the study was independently extracted by two reviews. Briefly, we extracted the name of the first author, the publication year, the number of cases and control, genotype of cases and control, country of study and genotyping methods.

### Statistical analysis

Hardy–Weinberg equilibrium (HWE) was applied for sample selection bias by using Chi-square test to identify low quality in individual study before pooling. The strength of association between TLR4 polymorphisms and hepatitis C infection were represented as OR with 95% confident intervals. All allelic models (Allele contrast, homozygous comparison, heterozygous comparison, dominant model, recessive model and over-dominant model) were examined for association by using an adjusted *P*-value for multiple testing via Bonferroni method. The heterogeneous studies were assessed by *I*^2^ value and Cochran Q test. *I*^2^ with <50% was considered a homogeneous population. Consequently, the pool OR was combined using the fixed-effect model or random effect model depending on the homogeneous population. Moreover, publication bias was tested by funnel plot and Egger’s regression test. Due to the fact that rs2149356 has a limited amount of evidence, we cannot calculate Egger’s regression for this allele. Thus, meta-analysis was performed using MetaGenyo [25].

### *In silico* prediction of precursor microRNA (miRNA), mature miRNA and target genes in rs2149356 sequence

The chromosome 9 GRGh38p12 sequence was downloaded from NCBI accession NC_000009.12. The TLR4 gene sequence was identified and used to search for the rs2149356 position. The gene was analyzed using miRNAFold to identify potential microRNA (miRNA) precursor in the comparison between T and G nucleotides [26,27]. Verified feature was 85% and sliding window site was 150 base pairs. To avoid a non-miRNA hairpin-like structure, we also confirmed the result via miRNABoost, in which the delta parameter was 0.25 [28]. The secondary structure was reconstructed by Forna [29]. Consequently, the pre-miRNA sequence identified the miRNA position by ab initio human pre-miRNA and miRNA prediction by hidden Markov model (HMM) algorithms [30]. Finally, the miRNA target analysis was reconstructed from psRNATarget by using Homo sapiens database selection [31]. The target genes of miRNA were functionally annotated via DAVID with high stringent criteria and represented as top sixth enrichment scores [32,33].

## Results

### Literature research and characteristics of included studies

**Figure 1:**
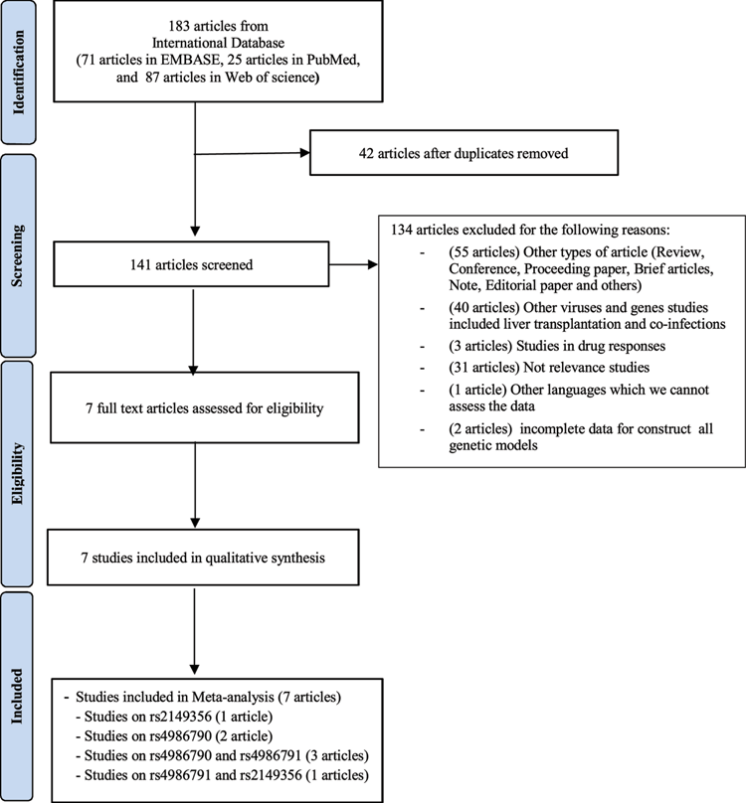
Flow diagram of selection for eligible studies enrolled in this meta-analysis

The databases (EMBASE, PubMed and Web of Science) showed a total of 141 publications relating to TLR4 polymorphisms and HCV after duplicated articles were removed. Thereafter, 134 articles were removed based on those containing unrelated titles, abstracts and full text, resulting in 7 eligible articles. Accordingly, only seven eligible articles were enrolled in this study for quality assessment and meta-analysis (Figure 1) [17–20,22,23,34]. Subsequently, quality assessment was performed via NOS and included studies were shown to be of high and moderate quality (Supplemental Table 1). Among these studies, the TLR4 polymorphisms in our meta-analysis were divided into rs4986791 in four studies (855 cases and 1305 controls), rs4986790 in five studies (1120 cases and 1420 controls) and rs2149356 in two studies (391 cases and 430 controls). Two studies determined the TLR4 polymorphisms via polymerase chain reaction-restriction fragment length polymorphism (PCR-RFLP) method. The characteristics and genotypes variation were summarized in Table 1. The genotype distributions in the controls were accessed through HWE; three studies (two studies in rs4986791 and one study in rs2149356) did not agree with HWE.

**Table 1:** Genotype distributions of HCV infected patient and healthy control

First authors	Cases			Controls			Methods	Country	HWE P-value*
	11^†^	12^†^	22^†^	11^†^	12^†^	22^†^			
rs4986791									
Agundez et al., 2012	274	33	1	341	47	2	Realtime PCR	Spain	0.79
Álvarez-Rodríguez et al., 2012	22	3	0	14	2	0	PCR-RFLP	Spain	0.79
Al-Qahtani et al., 2014	418	32	0	469	130	1	Realtime PCR	Arabia	0.01
Pires-Neto et al., 2015	68	4	0	276	22	1	Realtime PCR	Brazil	0.44
Total	782	72	1	1100	201	4			
rs4986790									
Álvarez-Rodríguez et al., 2012	22	3	0	14	2	0	PCR-RFLP	Spain	0.79
Al-Qahtani et al., 2014	408	41	0	475	123	2	Realtime PCR	Arabia	0.04
Pires-Neto et al., 2015	67	5	0	276	22	1	Realtime PCR	Brazil	0.44
Iqbal et al., 2017	232	116	52	52	79	14	Realtime PCR	Pakistan	0.04
Sghaier et al., 2018	47	52	75	253	77	30	PCR-RFLP	Tunisia	0.00
Total	776	217	127	1070	303	47			
rs2149356									
Agundez et al., 2012	28	96	184	28	176	186	Realtime PCR	Spain	0.12
Sadik et al., 2015	34	48	1	6	30	4	Realtime PCR	Egypt	0.00
Total	62	144	185	34	206	190			

* *P-value* of HWE was calculated by Chi-square test.

† 11, 12, 22: CC, CT and TT for rs 4986791; AA, AG and GG for rs4986790; TT, TG and GG of rs2149356.

### Synthesis, publication bias and sensitivity analysis

Meta-analysis of rs4986791 involved in four studies showed there were no significant associations in any genetic models between TLR4 polymorphisms and HCV infection (Table 2). In the over-dominant model, OR and 95% CI was 0.5781 (0.26, 3.6) under high heterogeneity (*I*^2^ = 79%, for example). Publication bias was assessed by using both the Egger’s test and funnel plot (Supplementary Figure 1A). Egger’s test showed no obvious publication bias (*P=*0.6104). Thereby, subgroups analysis was performed via excluded non-HWE study. However, the results were similar to pooled analysis, which showed no statistically significant association (Data not shown).

**Table 2:** Meta-analysis of association between TLR4 polymorphism and HCV infection

Genetic models	Models*	OR (95% CI)	*P-value*^†^	*I*^2^ (%)	*P-value*^‡^
rs4986791					
C vs T (Allele contrast)	RE	1.74 (0.84;3.60)	0.14	77.1	0.005
CC vs TT (Homozygous)	FE	1.50 (0.29;7.82)	<0.0001	0.0	0.856
CT vs TT (Heterozygous)	FE	0.95 (0.18;5.14)	0.96	0.0	0.909
CC vs CT (Heterozygous)	RE	1.73 (0.78;3.83)	0.17	79.0	0.003
CC+CT vs TT (Dominant model)	FE	1.42 (0.27;7.37)	0.68	0.0	0.881
CC vs CT + TT (Recessive model)	RE	1.76 (0.08;3.88)	0.16	79.1	0.003
CT vs CC+TT (Overdominant model)	RE	0.58 (0.26;1.28)	0.18	79.0	0.003
rs4986790					
A vs G (Allele contrast)	RE	0.94 (0.26;3.37)	<0.0001	97.7	<0.001
AA vs GG (Homozygous)	RE	0.58 (0.07;4.77)	0.61	93.4	<0.001
AG vs GG (Heterozygous)	FE	0.33 (0.21;0.49)	<0.0001	0.0	0.550
AA vs AG (Heterozygous)	RE	1.21 (0.41;3.56)	0.72	94.3	<0.001
AA+AG vs GG (Dominant model)	RE	0.47 (0.10;2.08)	0.32	87.2	<0.001
AA vs AG + GG (Recessive model)	RE	1.03 (0.26;4.00)	0.96	96.8	<0.001
AG vs AA+GG (Overdominant model)	RE	0.67 (0.31;1.41)	0.29	88.5	<0.001
rs2149356					
T vs G (Allele contrast)	RE	1.23 (0.47;3.28)	0.67	90.6	0.001
TT vs GG (Homozygous)	RE	3.84 (0.19;78.57)	0.38	84.2	0.012
TG vs GG (Heterozygous)	RE	1.45 (0.14;15.14)	0.76	77.9	0.034
TT vs TG (Heterozygous)	FE	2.17 (1.32;3.58)	0.00	22.1	0.257
TT+TG vs GG (Dominant model)	RE	1.87 (0.14;25.24)	0.64	81.9	0.019
TT vs TG + GG (Recessive model)	RE	2.09 (0.71;6.16)	0.18	73.8	0.051
TG vs TT+GG (Overdominant model)	FE	0.54 (0.40;0.75)	<0.0001	0.0	0.684

* Meta-analysis models; FE, fixed-effect model; RE, random effect model .

† OR *P*-value.

‡ heterogeneous *P*-value.

**Figure 2:**
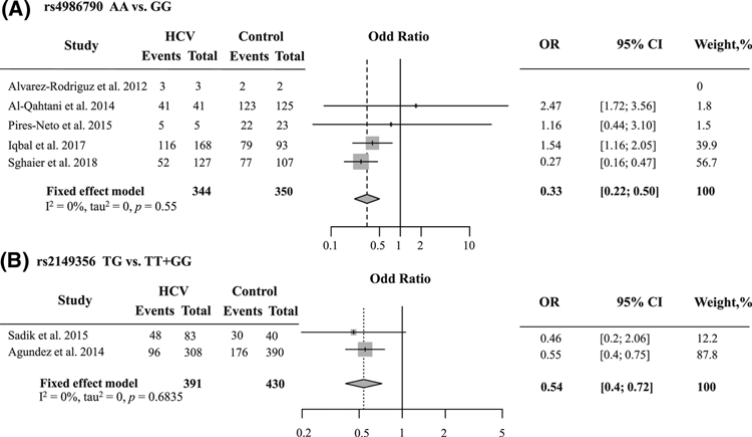
Forest plot of association between two TLR4 polymorphisms and HCV infection The area of the square was proportional to the study’s weight. The horizontal line represents 95% CI. The overall effect is illustrated as diamonds with the lateral points showing CI. (**A**) The forest plot of rs4986790 is shown as the heterozygous model. (**B**) The forest plot of rs2149356 is shown as the over-dominant model.

For rs4986790, the pooled analysis showed heterozygous model (AG vs GG: OR = 0.33, 95% CI = 0.21–0.49, *P*<0.0001 (Figure 2A)), significantly resistant to HCV infection. Egger’s statistics showed no publication bias (*P*=0.0768). The funnel plot appeared to be symmetrical (Supplementary Figure 1B). As aforementioned result, there was a study that it was not agreed with HWE in a control group. Therefore, we applied sensitivity analysis by using sequentially omitting individual study to confirm our meta-analysis (Supplementary Figure 2). The results did not change after excluded them. It suggested that this meta-analysis was stable.

Regarding rs2149356, two genetic models associated with HCV infection are shown in Table 2 (TT vs TG: OR = 2.17, 95% CI = 1.32–1.58, *P*=0.00; TG vs TT + GG: OR = 0.53, 95% CI = 0.40–0.72, *P*<0.0001 (Figure 2B)). The funnel plot was symmetrical (Supplementary Figure 1C). Because one study was not in HWE, we ignored this article and observed the OR. The result was not different (Supplementary Figure 3). Our finding indicated for the first time that the rs2149356 genotype TG might be a protective factor for HCV infection.

### *In silico* analysis of rs2149356 function as a siRNA

**Figure 3:**
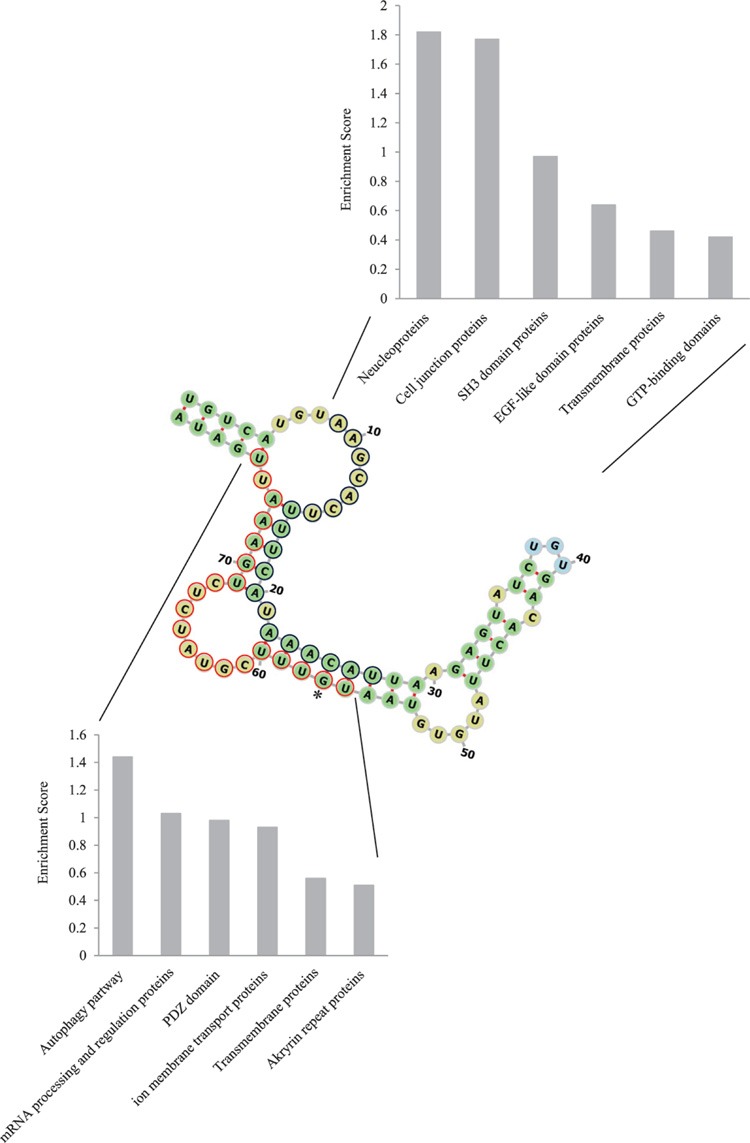
Pre-miRNA secondary structures and their target gene annotation The pre-miRNA structure was reconstructed via Forna. The stem loops are shown in green. The terminal step loop is shown in blue, while internal loops and bulges are shown in yellow. The navy and red cycle represent siRNA1 and siRNA2, respectively. The asterisk indicates rs2149356G location. The bar graph represents gene ontology group classified by DAVID.

The rs2149356 is located in the un-translational region at 7749 of the TLR4 gene. Therefore, a possible function of this position may relate to miRNA due to the fact that most miRNA sequences are located in the intergenic region or part of introns [35]. Our computational approach to identify rs2149356 as pre-miRNA showed that rs2149356G was able to form a secondary structure at 7691–7769, in which minimum free energy was −14.7 kcal/mol (Figure 3). On the contrary, we could not detect the secondary structure formation of rs2149356T. Furthermore, we validated this pre-miRNA by using a different algorithm, which showed 100% miRNA proportion. Additionally, this pre-miRNA was cleaved into two predicted miRNA (Figure 3). The first strand, called siRNA1, is located at 7699–7717 and other the strand, called siRNA2, at 7746–7766. Their targets were predicted (Supplementary Tables 2 and 3). Because miRNA plays a pivotal role in several biological processes, we analyzed the putative functions of our miRNA target using DAVID gene annotation (Supplementary Tables 4 and 5). Intriguingly, the nucleoproteins and proteins involved in the autophagy pathway were identified as the main targets of siRNA1 and siRNA2, respectively (Figure 3).

## Discussion

The TLR4 polymorphisms are proposed to associate with various diseases such as gastric cancer, prostate cancer, primary open-angle glaucoma and bacterial infection [36–38]. TLR4 is a pattern recognition receptor generally binding to LPS of Gram-negative bacteria. However, the roles of TLR4 have been explored in recent decades in terms of viral infection, especially flaviviridae (Flavivirus; Dengue and Zika virus and Hepecivirus; HCV). Dengue viral NS1 is able to activate TLR4 signaling via direct interaction [9]. Previously, the TLR4 expressions in peripheral blood mononuclear cells were shown an association between drug treatment and clinical outcomes of infection [39,40]. Moreover, HCV infection also closely relates to TLR4 as shown by various studies [14,16]. Therefore, TLR4 may play a crucial step in harmonization with other molecules for the elimination of HCV infection and restriction of chronic infections. Our study is the first meta-analysis to combine the shreds of evidence for association between three TLR4 polymorphisms (rs4986791, rs4986790 and rs2149356) and HCV infection.

In our meta-analysis, we could not detect any association between rs4986791 and HCV infection, suggesting that this position might not be a risk or protective factor. It is possible that the genotyping showed only one case of TT genotype which is a limitation in the genetic model calculation, such as the dominant model. To confirm these proposed, larger studies are required to increase genotype distribution. However, several studies have suggested that this polymorphism is involved in other biological processes of HCV evasion. The heterozygous and homozygous Asp299Gly/Thr399Ile (rs4986790 and rs4986791) polymorphisms are correlated with high viral loading and delayed response to viral therapy [15,16]. On the contrary with rs4986791, the rs4986790, which the AG genotype was a protective factor, is associated with HCV susceptibility via our meta-analysis. This was supported by Cussigh et al., who proposed that the rs4976790 was associated with liver fibrosis and influenced worse outcomes of HBV patients by using meta-analysis [41]. Interestingly, this mutation appears in the ligand-binding site of TLR4, but does not intrude on LPS binding [42]. Therefore, it is possible that this mutation may affect other properties of TLR4 between host and viral interaction.

Previous reports have suggested that rs2149356, located in the non-coding region, has functions under-investigated that are associated with the development of HHC. The TLR4 rs2149356 T allele reduces the risk of HHC compared with HCV patients and healthy controls [22]. Moreover, TT genotype frequency increased in responder patients versus non-responders to peg-IFN-α2b-ribavirin [23]. In our study, we assessed different questions from previous reports. We revealed that the TG genotype was a protective factor for HCV infection. In addition, we could not detect any association between T allele and HCV infection in the allele contrast model, suggesting that the T allele may not be related to HCV infection. However, our *in silico* analysis suggested that G allele was able to produce miRNA, in which the main functional annotation of targets showed the highest enrichment in the autophagy pathway. This is interesting since HCV induces autophagy to inhibit host innate immunity and cell death [43,44]. We speculated that rs2149356 TG may alter the autophagy pathway and causes a limitation of HCV infection. Further experimentation to investigate the role of rs2149356 is required.

Our study possesses two hallmarks that are different from previous studies. First, we combined the HHC and chronic HCV as a patient group, including responder and non-responder patients, to compare with healthy control. Therefore, our results were able to represent protective genotypes to HCV infection. Second, our meta-analysis incorporated two studies of rs214956 to increase population size. Altogether with previous studies, we postulated that rs2149356 plays an essential role during HCV-host interaction. TG genotype protected the HCV infection and TT genotype increased the success rate of HCV therapy via peg-IFN-α2b-ribavirin treatment. Finally, T allele of this SNP decreased the risk of HHC.

The inadequacies and restrictions existent in this study are significant and should be taken into account for subsequent research concerning similar topics. First, this study only merged seven previous reports concerning HCV infection, including two studies for rs2149356: five analyses of rs4986790 and four investigations of rs4986791. The individual studies carried on small samples which may decrease the power of meta-analysis, especially rs2149356 composed of 391 cases and 430 controls. There are some studies in our meta-analysis that are not based on the HWE. Therefore, in order to enhance the validity of the link with HCV infection and genotype distribution, future studies should consider conducting research employing a larger sampling comprised of a variety of ethnic groups. Additionally, research concerning rs2149356 and rs4986790 should exhibit a comparatively enhanced sample size. Thus, additional research employing a more expansive sample size should be involved as a way to improve validity. Besides, the genotyping methods in our meta-analysis are heterogeneity between realtime-PCR and PCR-RFLP which may affect our results. Several original articles performed the genotyping via only one method, which should be confirmed by others, and did not calculate call rate. Furthermore, additional elements may influence the results of research, such as supplementary gene polymorphisms. Our study could not assess the haplotype between HCV patients and healthy controls. Additionally, we did not analyze the association of TLR4 polymorphisms with other outcomes of HCV infection such as fibrosis, drug treatment and HCC due to lack of sufficient data for eligible analysis.

In summary, the studies of TLR4 polymorphisms and HCV are still required in several aspects to verify these phenomena in an epidemiological effort. Especially, the TLR4 gene polymorphisms should be compared between persistence infection and spontaneous viral clearance due to the fact that uncommon cases are self-limitation which occurs approximately 20% [45]. The understanding of this factor may be applied to combat HCV infection.

## Conclusion

We found the association between TLR polymorphisms and HCV infection from collected publications in related topics, although our study did not show strong evidence due to insufficient material. Our result implied that the rs4986790 decreased a risk of infection with HCV. The rs2149356 TG genotype was a protective factor via the possible mechanism relating to miRNA, which regulated the autophagy partway. This knowledge was rediscovered from a systematic review, meta-analysis and *in silico* prediction. Our study and results may be applied and considered in planning for personal and precise medicine to eradicate HCV.

## Supporting information

**Supplementary Figure 1:** Funnel plot of the included study

**Supplementary Table 1:** The NOS analysis of inclued stuies

**Supplementary Table 2:** List of miRNA2 target prediction

**Supplementary Table 3:** List of miRNA2 target prediction

**Supplementary Table 4:** List of siRNA1 target gene annotation by DAVID

**Supplementary Table 5:** List of siRNA2 target gene annotation by DAVID

## Abbreviations

CI: confidence interval
HCV: hepatitis C virus
HHC: HCV-induced hepatocellular carcinoma
HWE: Hardy–Weinberg equilibrium
LPS: lipopolysaccharides
miRNA: micro RNA
NOS: Newcastle–Ottawa scale
OR: odd-ratio
PCR-RFLP: polymerase chain reaction-restriction fragment length polymorphism
SNP: single nucleotide polymorphism
TLR: Toll-like receptor
