# Supplementary material for: The associations between Toll-like receptor 4 gene polymorphisms and hepatitis C virus infection: a systematic review and meta-analysis
Source: Biosci Rep. 2019 Feb 26;39(2):BSR20182470. doi: 10.1042/BSR20182470 (PMC6390129; doi:10.1042/BSR20182470)
Supplement: Supplementary file 1 [file bsr-39-bsr20182470_Supp1.pdf]

A. rs4986791

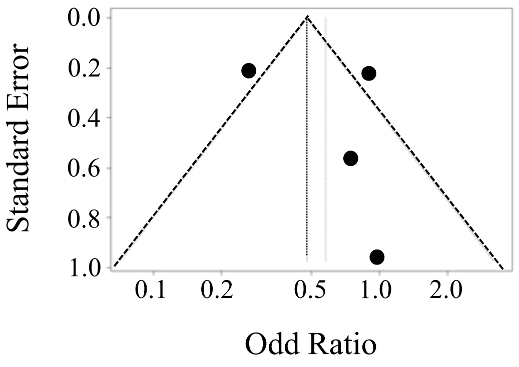

B. rs4986790

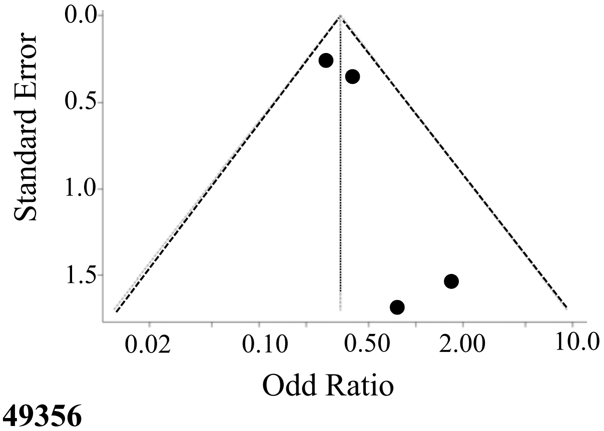

C. rs2149356

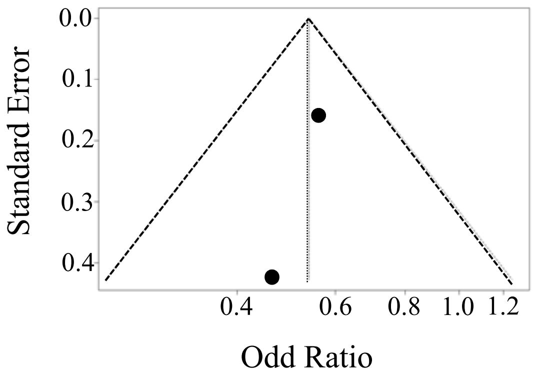

Supplementary Figure1 Funnel plot of the included study

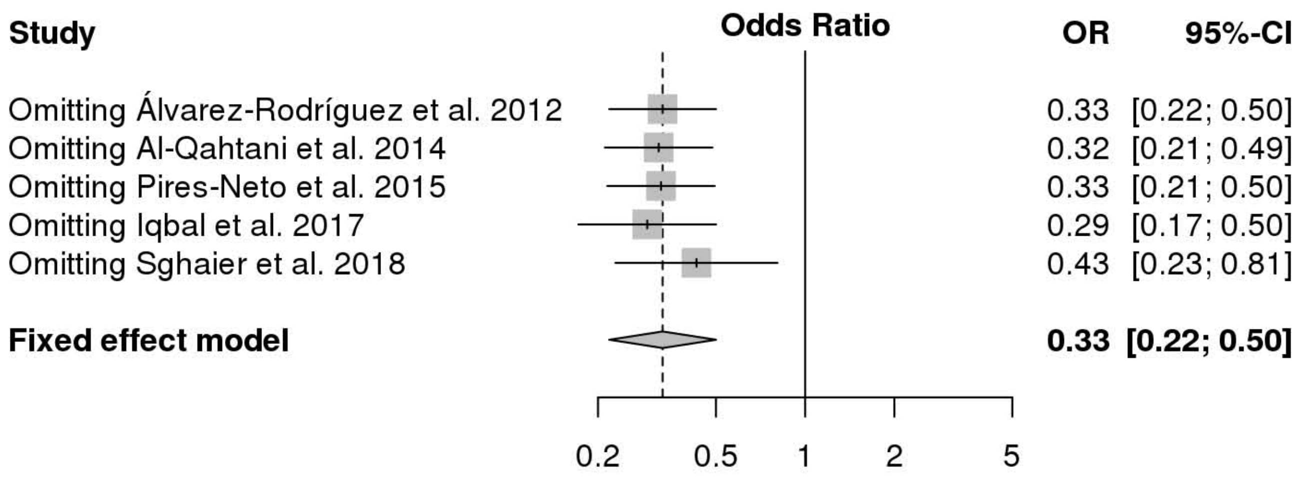
Study
Odds Ratio
OR
95%-CI
Omitting Álvarez-Rodríguez et al. 2012
Omitting Al-Qahtani et al. 2014
Omitting Pires-Neto et al. 2015
Omitting Iqbal et al. 2017
Omitting Sghaier et al. 2018
Fixed effect model
0.33 [0.22; 0.50]
0.32 [0.21; 0.49]
0.33 [0.21; 0.50]
0.29 [0.17; 0.50]
0.43 [0.23; 0.81]
0.33 [0.22; 0.50]
0.2
0.5
1
2
5

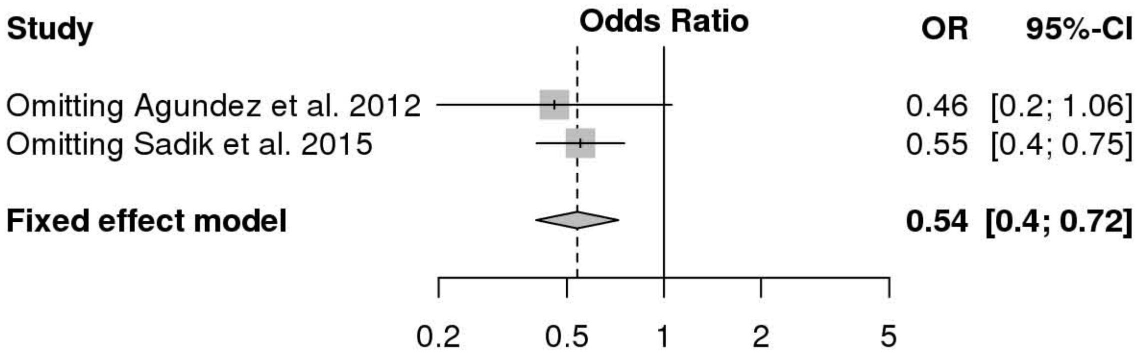
Study
Odds Ratio
OR
95%-CI
Omitting Agundez et al. 2012
0.46 [0.2; 1.06]
Omitting Sadik et al. 2015
0.55 [0.4; 0.75]
Fixed effect model
0.54 [0.4; 0.72]
0.2
0.5
1
2
5

Supplementary Table1 The NOS analysis of inclued stuies

**Study ID**	Sadik et al. 2015	Pires-Neto et al. 2015	Álvarez-Rodríguez et al. 2012	Al-Qahtani et al. 2014	Agundez et al. 2012	Lqbal et al.2017
**Selection**						
Is the case definition adequate?	Serum HCV RNA positive (a*)	HCV antibody and Serum HCV RNA positive (a*)	HCV infection and treatmented with antiviral drug (a*)	HCV antibody and Serum HCV RNA positive (a*)	positive HCV viremia (a*)	Both ELISA and PCR positive (a*)
Representativeness of the cases	consecutive cases (a*)	not stated	consecutive cases (a*)	not stated	consecutive cases (a*)	consecutive cases (a*)
Selection of Controls	hospital controls and genetic polymophisms of interest in HWE	community controls and genetic polymophisms of interest in HWE (a*)	community controls and genetic polymophisms of interest in HWE (a*)	hospital controls and genetic polymophisms of interest in HWE	no description	no description
Definition of Controls	Seological negative (a*)	Seological negative (a*)	healthy control (a*)	Seological negative and PCR negative (a*)	healthy volunteers/ standard clinical and anlytical procedures (a*)	healthy control (a*)
**Comparability**						
cases and controls of homogeneous ethnic descent	may heterogenous population between cases and control	may heterogenous population between cases and control	may heterogenous population between cases and control	Homogenous ethinic descent (a*)	Homogenous ethinic descent (a*): caucasian	may heterogenous population between cases and control
no evidence of population stratification	Epyptian (a*)	no evidence	no evidence	Saudi origin (a*)	stratification	no evidence
**Exposure**						
Ascertainment of exposure	no report of quality control precedures or blinding	no report of quality control precedures or blinding	no report of quality control precedures or blinding	no report of quality control precedures or blinding	triplicated samples and duplicate analysis but not report blinding	no report of quality control precedures or blinding
Same method of ascertainment for cases and controls	Yes (a*)	Yes (a*)	Yes (a*)	Yes (a*)	Yes (a*)	Yes (a*)
Genotyping call rate	Not reported	Not reported	Not reported	Not reported	Not reported	Not reported
Total	5	4	4	5	5	4

Sghaier et al., 2018
HCV antibody and Serum HCV RNA positive (a*)
consecutive cases (a*)
no description
Seological negative (a*)
may heterogenous population between cases and control
no evidence
no report of quality control precedures or blinding
Yes (a*)
Not reported
4

Supplementary Table 2 List of miRNA2 target prediction				
miRNA_Acc.	Target_Acc.	Expectation	Target_start	Target_end
aagcacuuuucauaaacau	NM_001163152|ETV1	2	2793	2811
aagcacuuuucauaaacau	NM_014213|HOXD9	2.5	341	359
aagcacuuuucauaaacau	NM_152280|SYT11	3	3304	3322
aagcacuuuucauaaacau	NM_000209|PDX1	3	1475	1493
aagcacuuuucauaaacau	NM_001005473|PLCXD3	3	1915	1933
aagcacuuuucauaaacau	NM_001190942|TNFSF1	3	207	225
aagcacuuuucauaaacau	NM_015576|ERC2	3	2935	2953
aagcacuuuucauaaacau	NM_001256105|WNT5A	3.5	2275	2293
aagcacuuuucauaaacau	NM_004675|DIRAS3	3.5	407	425
aagcacuuuucauaaacau	NM_138794|LYPLAL1	3.5	36	54
aagcacuuuucauaaacau	NM_152495|CNIH3	3.5	1129	1147
aagcacuuuucauaaacau	NM_198513|PHF20L1	3.5	1228	1246
aagcacuuuucauaaacau	NM_020700|PPM1H	3.5	1896	1914
aagcacuuuucauaaacau	NM_025142|TAOK1	3.5	6377	6395
aagcacuuuucauaaacau	NM_019050|USP53	3.5	1176	1194
aagcacuuuucauaaacau	NM_001198952|RNF103	3.5	733	751
aagcacuuuucauaaacau	NM_001144073|CHORD	3.5	1937	1955
aagcacuuuucauaaacau	NM_001106|ACVR2B	3.5	8727	8745
aagcacuuuucauaaacau	NM_001191005|SRSF10	4	1651	1669
aagcacuuuucauaaacau	NM_001193628|SMIM17	4	1508	1526
aagcacuuuucauaaacau	NM_013943|CLIC4	4	2359	2377
aagcacuuuucauaaacau	NM_032504|UNC80	4	3616	3634
aagcacuuuucauaaacau	NM_006775|QKI	4	2355	2373
aagcacuuuucauaaacau	NM_206853|QKI	4	7094	7112
aagcacuuuucauaaacau	NM_206854|QKI	4	8355	8373
aagcacuuuucauaaacau	NM_206855|QKI	4	9320	9338
aagcacuuuucauaaacau	NM_001025290|DPPA5	4	189	207
aagcacuuuucauaaacau	NM_206832|TMIGD1	4	167	185
aagcacuuuucauaaacau	NM_020937|FANCM	4	230	248
aagcacuuuucauaaacau	NM_001039152|RGS21	4	478	496
aagcacuuuucauaaacau	NM_001174159|SH2D4A	4	717	735
aagcacuuuucauaaacau	NM_020698|TMCC3	4	1508	1526
aagcacuuuucauaaacau	NM_020949|SLC7A14	4	7019	7038
aagcacuuuucauaaacau	NM_005188|CBL	4	3034	3052
aagcacuuuucauaaacau	NM_015398|FAM149A	4	509	527
aagcacuuuucauaaacau	NM_017759|INO80D	4	7818	7836
aagcacuuuucauaaacau	NM_005882|MAEA	4	225	243
aagcacuuuucauaaacau	NM_031435|THAP2	4	234	252
aagcacuuuucauaaacau	NM_133445|GRIN3A	4	3603	3621
aagcacuuuucauaaacau	NM_152754|SEMA3D	4	3645	3663
aagcacuuuucauaaacau	NM_014728|FRMPD4	4	3414	3432
aagcacuuuucauaaacau	NM_001128|AP1G1	4	2386	2404
aagcacuuuucauaaacau	NM_001040151|SCN3B	4	4054	4072
aagcacuuuucauaaacau	NM_002264|KPNA1	4	5053	5071
aagcacuuuucauaaacau	NM_002336|LRP6	4	3434	3452
aagcacuuuucauaaacau	NM_001001420|SMAD5	4	3975	3993
aagcacuuuucauaaacau	NM_173348|FAM149B1	4	106	124
aagcacuuuucauaaacau	NM_001995|ACSL1	4	1086	1104
aagcacuuuucauaaacau	NM_001195556|CLINT1	4	1609	1627
aagcacuuuucauaaacau	NM_004567|PFKFB4	4	342	360
aagcacuuuucauaaacau	NM_001010853|PM20D2	4.5	2541	2559
aagcacuuuucauaaacau	NM_001198681|LEPROT	4.5	730	748
aagcacuuuucauaaacau	NM_015221|DNMBP	4.5	1497	1515
aagcacuuuucauaaacau	NM_005160|ADRBK2	4.5	2189	2207
aagcacuuuucauaaacau	NM_018700|TRIM36	4.5	1190	1208

aagcacuuuucauaaacau	NM_020403|PCDH9	4.5	1694	1712
aagcacuuuucauaaacau	NM_172070|UBR3	4.5	2266	2284
aagcacuuuucauaaacau	NM_003559|PIP4K2B	4.5	2074	2092
aagcacuuuucauaaacau	NM_005842|SPRY2	4.5	274	292
aagcacuuuucauaaacau	NM_052870|SNX18	4.5	356	374
aagcacuuuucauaaacau	NM_014363|SACS	4.5	722	740
aagcacuuuucauaaacau	NM_001270410|LIN9	4.5	34	52
aagcacuuuucauaaacau	NM_152418|DCAF4L2	4.5	778	796
aagcacuuuucauaaacau	NM_001191029|C17orf72	4.5	1697	1715
aagcacuuuucauaaacau	NM_001171197|ELAVL2	4.5	458	476
aagcacuuuucauaaacau	NM_001191030|C17orf72	4.5	1911	1929
aagcacuuuucauaaacau	NM_001191031|C17orf72	4.5	1957	1975
aagcacuuuucauaaacau	NM_005012|ROR1	4.5	2616	2634
aagcacuuuucauaaacau	NM_004232|SOCS6	4.5	991	1009
aagcacuuuucauaaacau	NM_014746|RNF144A	4.5	433	451
aagcacuuuucauaaacau	NM_016354|SLCO4A1	4.5	232	250
aagcacuuuucauaaacau	NM_145866|FZD3	4.5	10964	10982
aagcacuuuucauaaacau	NM_001018025|MTCP1	4.5	1226	1244
aagcacuuuucauaaacau	NM_001170588|HHAT	4.5	956	974
aagcacuuuucauaaacau	NM_001124756|PABPC1	4.5	170	188
aagcacuuuucauaaacau	NM_004274|AKAP6	4.5	468	486
aagcacuuuucauaaacau	NM_001010883|FAM102	4.5	2767	2785
aagcacuuuucauaaacau	NM_004315|ASAH1	4.5	929	947
aagcacuuuucauaaacau	NM_030762|BHLHE41	4.5	2002	2020
aagcacuuuucauaaacau	NM_020714|ZNF490	4.5	2421	2439
aagcacuuuucauaaacau	NM_001159643|MCTP2	4.5	3639	3657
aagcacuuuucauaaacau	NM_001031701|NT5DC3	4.5	1864	1882
aagcacuuuucauaaacau	NM_015339|ADNP	4.5	321	339
aagcacuuuucauaaacau	NM_001999|FBN2	4.5	1140	1158
aagcacuuuucauaaacau	NM_001168271|GPR156	4.5	822	840
aagcacuuuucauaaacau	NM_006865|LILRA3	4.5	129	147
aagcacuuuucauaaacau	NM_181744|OPN5	4.5	9	27
aagcacuuuucauaaacau	NM_001013743|TMEM2	4.5	32	50
aagcacuuuucauaaacau	NM_006981|NR4A3	4.5	2855	2873
aagcacuuuucauaaacau	NM_014425|INVS	4.5	232	250
aagcacuuuucauaaacau	NM_000456|SUOX	4.5	365	383
aagcacuuuucauaaacau	NM_017593|BMP2K	4.5	46	64
aagcacuuuucauaaacau	NM_004234|ZNF235	4.5	69	87
aagcacuuuucauaaacau	NM_001135703|LRP12	4.5	1241	1259
aagcacuuuucauaaacau	NM_001007226|SPOP	4.5	516	534
aagcacuuuucauaaacau	NM_004520|KIF2A	4.5	95	113
aagcacuuuucauaaacau	NM_001105568|KIF13A	4.5	416	434
aagcacuuuucauaaacau	NM_001198615|MAP7	4.5	1182	1200
aagcacuuuucauaaacau	NM_015289|VPS39	4.5	1141	1159
aagcacuuuucauaaacau	NM_005863|NET1	4.5	260	278
aagcacuuuucauaaacau	NM_006489|NOVA1	4.5	227	245
aagcacuuuucauaaacau	NM_006999|PAPD7	4.5	2047	2065
aagcacuuuucauaaacau	NM_018141|MRPS10	4.75	56	76
aagcacuuuucauaaacau	NM_006775|QKI	5	7120	7137
aagcacuuuucauaaacau	NM_198461|LONRF2	5	7177	7195
aagcacuuuucauaaacau	NM_206853|QKI	5	11859	11876
aagcacuuuucauaaacau	NM_020742|NLGN4X	5	1166	1185
aagcacuuuucauaaacau	NM_004232|SOCS6	5	3560	3578
aagcacuuuucauaaacau	NM_001040653|ZXDC	5	7070	7090
aagcacuuuucauaaacau	NM_001080477|TENM3	5	722	740
aagcacuuuucauaaacau	NM_001007094|ZNF37A	5	4342	4360
aagcacuuuucauaaacau	NM_020183|ARNTL2	5	4397	4415

aagcacuuuucauaaacau	NM_001248005|ARNTL2	5	4465	4483
aagcacuuuucauaaacau	NM_021072|HCN1	5	329	347
aagcacuuuucauaaacau	NM_022658|HOXC8	5	1204	1222
aagcacuuuucauaaacau	NM_015234|GPR116	5	1382	1400
aagcacuuuucauaaacau	NM_181670|ANKS1B	5	983	1001
aagcacuuuucauaaacau	NM_016626|MEX3C	5	372	390
aagcacuuuucauaaacau	NM_001204068|ANKS1B	5	1077	1095
aagcacuuuucauaaacau	NM_152305|POGLUT1	5	479	497
aagcacuuuucauaaacau	NM_001077637|WTH3D	5	2381	2399
aagcacuuuucauaaacau	NM_003412|ZIC1	5	773	791
aagcacuuuucauaaacau	NM_001037553|AGPAT1	5	4685	4703
aagcacuuuucauaaacau	NM_182543|NSUN6	5	374	393
aagcacuuuucauaaacau	NM_001999|FBN2	5	488	506
aagcacuuuucauaaacau	NM_001009555|SH3D19	5	1268	1286
aagcacuuuucauaaacau	NM_000578|SLC11A1	5	1490	1508
aagcacuuuucauaaacau	NM_014705|DOCK4	5	1666	1684
aagcacuuuucauaaacau	NM_003930|SKAP2	5	1524	1542
aagcacuuuucauaaacau	NM_198935|SS18L1	5	2601	2619
aagcacuuuucauaaacau	NM_052907|TMEM132B	5	1767	1785
aagcacuuuucauaaacau	NM_000092|COL4A4	5	1609	1627
aagcacuuuucauaaacau	NM_001077199|SREK1	5	4169	4187
aagcacuuuucauaaacau	NM_007007|CPSF6	5	1668	1686
aagcacuuuucauaaacau	NM_001127715|STXBP5	5	5617	5635
aagcacuuuucauaaacau	NM_001127715|STXBP5	5	2941	2959
aagcacuuuucauaaacau	NM_001190965|ZMYM2	5	2410	2428
aagcacuuuucauaaacau	NM_001017977|DCAF6	5	217	235
aagcacuuuucauaaacau	NM_001114394|PAPD4	5	1076	1094
aagcacuuuucauaaacau	NM_018120|ARMC1	5	691	709
aagcacuuuucauaaacau	NM_001037174|ARL5A	5	422	443
aagcacuuuucauaaacau	NM_001174129|SLC11A	5	2086	2104

Supplementary Table 3 List of miRNA2 target prediction				
miRNA_Acc.	Target_Acc.	Expectation	Target_start	Target_end
uguuucguaucucugaaauu	NM_144968|RIBC1	3	913	932
uguuucguaucucugaaauu	NM_001144935|FGF1	3	2183	2202
uguuucguaucucugaaauu	NM_033136|FGF1	3	2364	2383
uguuucguaucucugaaauu	NM_017563|IL17RD	3	4302	4321
uguuucguaucucugaaauu	NM_015836|WARS2	3	587	606
uguuucguaucucugaaauu	NM_201263|WARS2	3	1036	1055
uguuucguaucucugaaauu	NM_001164232|DDHD2	3.5	1572	1591
uguuucguaucucugaaauu	NM_021956|GRIK2	3.5	1001	1020
uguuucguaucucugaaauu	NM_001166247|GRIK2	3.5	1181	1200
uguuucguaucucugaaauu	NM_175768|GRIK2	3.5	1205	1224
uguuucguaucucugaaauu	NM_001256021|TRDN	3.5	217	236
uguuucguaucucugaaauu	NM_176875|CCKBR	3.5	51	70
uguuucguaucucugaaauu	NM_003766|BECN1	3.5	531	550
uguuucguaucucugaaauu	NM_006773|DDX18	3.5	169	188
uguuucguaucucugaaauu	NM_033089|ZCCHC3	3.5	1019	1038
uguuucguaucucugaaauu	NM_032558|HIATL1	4	1224	1243
uguuucguaucucugaaauu	NM_174909|TMEM167A	4	1145	1164
uguuucguaucucugaaauu	NM_001326|CSTF3	4	166	184
uguuucguaucucugaaauu	NM_198461|LONRF2	4	10477	10496
uguuucguaucucugaaauu	NM_016200|NAA38	4	8254	8273
uguuucguaucucugaaauu	NM_152321|ERP27	4	64	83
uguuucguaucucugaaauu	NM_001163315|FBXL17	4	929	948
uguuucguaucucugaaauu	NM_005364|MAGEA8	4	430	449
uguuucguaucucugaaauu	NM_016357|LIMA1	4	1189	1208
uguuucguaucucugaaauu	NM_004234|ZNF235	4	184	203
uguuucguaucucugaaauu	NM_001033723|ZNF704	4	856	875
uguuucguaucucugaaauu	NM_001258247|TMEM21	4	2965	2984
uguuucguaucucugaaauu	NM_001199563|BVES	4	2269	2288
uguuucguaucucugaaauu	NM_030923|TMEM163	4	676	695
uguuucguaucucugaaauu	NM_017761|PNRC2	4	1052	1071
uguuucguaucucugaaauu	NM_001206889|CTNND1	4	2387	2406
uguuucguaucucugaaauu	NM_015332|NUDCD3	4	3578	3597
uguuucguaucucugaaauu	NM_004663|RAB11A	4	1138	1157
uguuucguaucucugaaauu	NM_001206836|RAB11A	4	1226	1245
uguuucguaucucugaaauu	NM_001025266|C3orf70	4	3505	3524
uguuucguaucucugaaauu	NM_001003665|C1orf95	4	3906	3925
uguuucguaucucugaaauu	NM_006933|SLC5A3	4	1281	1300
uguuucguaucucugaaauu	NM_030799|YIPF5	4.5	862	881
uguuucguaucucugaaauu	NM_173666|DTWD2	4.5	3380	3399
uguuucguaucucugaaauu	NM_006774|INMT	4.5	1102	1121
uguuucguaucucugaaauu	NM_001163335|SYTL5	4.5	1462	1481
uguuucguaucucugaaauu	NM_007210|GALNT6	4.5	1177	1196
uguuucguaucucugaaauu	NM_139015|SPPL3	4.5	449	468
uguuucguaucucugaaauu	NM_194071|CREB3L2	4.5	4698	4717
uguuucguaucucugaaauu	NM_153259|MCOLN2	4.5	297	317
uguuucguaucucugaaauu	NM_001007067|SDCBP	4.5	199	218
uguuucguaucucugaaauu	NM_177531|PKHD1L1	4.5	459	478
uguuucguaucucugaaauu	NM_003253|TIAM1	4.5	624	643
uguuucguaucucugaaauu	NM_017946|FKBP14	4.5	236	255
uguuucguaucucugaaauu	NM_020390|EIF5A2	4.5	2406	2425
uguuucguaucucugaaauu	NM_016654|GABPB1	4.5	321	340
uguuucguaucucugaaauu	NM_001256368|LOC1001	4.5	743	762
uguuucguaucucugaaauu	NM_006460|HEXIM1	4.5	933	952
uguuucguaucucugaaauu	NM_015317|PUM2	4.5	1305	1324
uguuucguaucucugaaauu	NM_000314|PTEN	4.5	777	796

uguuucguaucucugaaauu	NM_004232|SOCS6	4.5	208	227
uguuucguaucucugaaauu	NM_001130700|IPCEF1	4.5	3910	3929
uguuucguaucucugaaauu	NM_173822|FAM126B	4.5	2068	2087
uguuucguaucucugaaauu	NM_000721|CACNA1E	4.5	5254	5273
uguuucguaucucugaaauu	NM_001178140|TFDP2	4.5	6288	6307
uguuucguaucucugaaauu	NM_138340|ABHD3	4.5	140	159
uguuucguaucucugaaauu	NM_019597|HNRNPH2	4.5	614	633
uguuucguaucucugaaauu	NM_001039763|TMEM23	4.5	516	535
uguuucguaucucugaaauu	NM_175873|SOWAHA	4.5	1276	1295
uguuucguaucucugaaauu	NM_020869|DCDC5	4.5	946	965
uguuucguaucucugaaauu	NM_001199974|RPL36A-	4.5	1960	1979
uguuucguaucucugaaauu	NM_007188|ABCB8	4.5	1367	1386
uguuucguaucucugaaauu	NM_001981|EPS15	4.5	517	536
uguuucguaucucugaaauu	NM_173495|PTCHD1	4.5	89	108
uguuucguaucucugaaauu	NM_018482|ASAP1	4.5	2283	2302
uguuucguaucucugaaauu	NM_002977|SCN9A	4.5	1443	1462
uguuucguaucucugaaauu	NM_015566|FAM169A	4.5	919	938
uguuucguaucucugaaauu	NM_002016|FLG	4.5	184	203
uguuucguaucucugaaauu	NM_024754|PTCD2	4.5	387	406
uguuucguaucucugaaauu	NM_001083535|CEP57L1	4.5	439	458
uguuucguaucucugaaauu	NM_001271853|CEP57L1	4.5	660	679
uguuucguaucucugaaauu	NM_198270|NHS	4.5	3374	3393
uguuucguaucucugaaauu	NM_001010922|BCL2L15	4.5	867	886
uguuucguaucucugaaauu	NM_001145399|MPPED2	4.5	1540	1559
uguuucguaucucugaaauu	NM_006738|AKAP13	4.5	634	653
uguuucguaucucugaaauu	NM_153607|CREBRF	4.5	283	302
uguuucguaucucugaaauu	NM_173814|PRTG	4.5	6095	6114
uguuucguaucucugaaauu	NM_033104|STON2	4.5	2910	2929
uguuucguaucucugaaauu	NM_001003845|SP5	4.5	347	366
uguuucguaucucugaaauu	NM_001276713|ANKDD1	4.5	768	787
uguuucguaucucugaaauu	NM_016607|ARMCX3	4.5	408	427
uguuucguaucucugaaauu	NM_006290|TNFAIP3	4.5	433	452
uguuucguaucucugaaauu	NM_002486|NCBP1	4.5	289	308
uguuucguaucucugaaauu	NM_001242798|ZNF322	4.5	2645	2664
uguuucguaucucugaaauu	NM_153251|ZDHHC20	4.5	2093	2112
uguuucguaucucugaaauu	NM_004519|KCNQ3	5	3777	3796
uguuucguaucucugaaauu	NM_003391|WNT2	5	979	998
uguuucguaucucugaaauu	NM_024430|PSTPIP2	5	963	982
uguuucguaucucugaaauu	NM_022156|DUS1L	5	223	242
uguuucguaucucugaaauu	NM_014805|EPM2AIP1	5	636	655
uguuucguaucucugaaauu	NM_007362|NCBP2	5	866	885
uguuucguaucucugaaauu	NM_001199492|PDCD4	5	892	911
uguuucguaucucugaaauu	NM_022041|GAN	5	2022	2041
uguuucguaucucugaaauu	NM_032811|TBRG1	5	58	77
uguuucguaucucugaaauu	NM_138973|BACE1	5	3776	3795
uguuucguaucucugaaauu	NM_024345|DCAF10	5	1923	1942
uguuucguaucucugaaauu	NM_001039547|GK5	5	5050	5069
uguuucguaucucugaaauu	NM_206855|QKI	5	55	74
uguuucguaucucugaaauu	NM_001098787|BET1L	5	2315	2333
uguuucguaucucugaaauu	NM_016526|BET1L	5	2483	2501
uguuucguaucucugaaauu	NM_015983|UBE2D4	5	626	644
uguuucguaucucugaaauu	NM_018176|LGI2	5	855	874
uguuucguaucucugaaauu	NM_012262|HS2ST1	5	3937	3955
uguuucguaucucugaaauu	NM_175698|SSX2	5	3	22
uguuucguaucucugaaauu	NM_001164417|SSX2B	5	3	22
uguuucguaucucugaaauu	NM_003147|SSX2	5	44	63
uguuucguaucucugaaauu	NM_001166395|CHST4	5	510	529

uguuucguaucucugaaauu	NM_014058|TMPRSS11E	5	59	78
uguuucguaucucugaaauu	NM_021927|GUF1	5	318	338
uguuucguaucucugaaauu	NM_001386|DPYSL2	5	1119	1138
uguuucguaucucugaaauu	NM_175566|CNTN5	5	2174	2193
uguuucguaucucugaaauu	NM_001256573|CHRNA4	5	2020	2039
uguuucguaucucugaaauu	NM_019116|UBFD1	5	76	95
uguuucguaucucugaaauu	NM_001164540|DISC1	5	93	112
uguuucguaucucugaaauu	NM_001080409|ZNF99	5	4270	4289
uguuucguaucucugaaauu	NM_020699|GATAD2B	5	2336	2355
uguuucguaucucugaaauu	NM_001169106|FAM21C	5	315	334
uguuucguaucucugaaauu	NM_002240|KCNJ6	5	654	673
uguuucguaucucugaaauu	NM_152549|CCDC112	5	419	438
uguuucguaucucugaaauu	NM_001166242|C22orf39	5	384	403
uguuucguaucucugaaauu	NM_153767|KCNJ1	5	74	93
uguuucguaucucugaaauu	NM_014394|GHITM	5	94	113
uguuucguaucucugaaauu	NM_024917|TRMT2B	5	1132	1151
uguuucguaucucugaaauu	NM_002850|PTPRS	5	822	841
uguuucguaucucugaaauu	NM_018105|THAP1	5	1263	1282
uguuucguaucucugaaauu	NM_001164416|H2BFM	5	275	294
uguuucguaucucugaaauu	NM_033103|RHPN2	5	257	276
uguuucguaucucugaaauu	NM_199003|THAP1	5	1547	1566
uguuucguaucucugaaauu	NM_001102608|COL6A6	5	957	976
uguuucguaucucugaaauu	NM_145756|ZNF396	5	1625	1644
uguuucguaucucugaaauu	NM_015842|LMO7	5	1611	1630
uguuucguaucucugaaauu	NM_005358|LMO7	5	1681	1700
uguuucguaucucugaaauu	NM_032569|GLYR1	5	1492	1511
uguuucguaucucugaaauu	NM_001135598|CLHC1	5	2864	2883
uguuucguaucucugaaauu	NM_014028|OSTM1	5	877	896
uguuucguaucucugaaauu	NM_001142673|ATG13	5	801	820
uguuucguaucucugaaauu	NM_001256141|FSBP	5	467	486
uguuucguaucucugaaauu	NM_018036|ATG2B	5	3803	3822
uguuucguaucucugaaauu	NM_005044|PRKX	5	3677	3696
uguuucguaucucugaaauu	NM_004376|COX15	5	3521	3540
uguuucguaucucugaaauu	NM_015026|MON2	5	772	791
uguuucguaucucugaaauu	NM_001205262|RAD54B	5	943	962

Supplementary Table 4 List of siRNA1 target gene annotation by DAVID				
	Enrichment Score: 1.82	Count	P_Value	Benjamini
INTERPRO	K Homology domain, type 1	4	3.10E-03	6.30E-01
INTERPRO	K Homology domain	3	2.60E-02	9.90E-01
SMART	KH	3	4.20E-02	9.80E-01
	Enrichment Score: 1.77	Count	P_Value	Benjamini
UP_KEYWORDS	Synapse	8	6.50E-03	4.60E-01
UP_KEYWORDS	Cell junction	10	2.30E-02	5.90E-01
GOTERM_CC_DIRECT	cell junction	8	3.20E-02	7.70E-01
	Enrichment Score: 1.2	Count	P_Value	Benjamini
UP_KEYWORDS	SH3 domain	5	4.30E-02	5.70E-01
INTERPRO	Src homology-3 domain	5	5.70E-02	9.80E-01
SMART	SH3	5	1.00E-01	9.90E-01
	Enrichment Score: 0.97	Count	P_Value	Benjamini
INTERPRO	RNA recognition motif domain	5	6.40E-02	9.50E-01
INTERPRO	Nucleotide-binding, alpha-beta plait	5	9.90E-02	9.80E-01
SMART	RRM	5	1.10E-01	9.70E-01
GOTERM_MF_DIRECT	nucleotide binding	5	2.00E-01	1.00E+00
	Enrichment Score: 0.64	Count	P_Value	Benjamini
UP_SEQ_FEATURE	domain:EGF-like 3	3	8.10E-02	1.00E+00
UP_SEQ_FEATURE	domain:EGF-like 2	3	1.10E-01	1.00E+00
UP_SEQ_FEATURE	domain:EGF-like 1	3	1.70E-01	1.00E+00
UP_KEYWORDS	EGF-like domain	3	4.30E-01	8.70E-01
INTERPRO	Epidermal growth factor-like domain	3	4.50E-01	1.00E+00
SMART	EGF	3	4.80E-01	1.00E+00
	Enrichment Score: 0.63	Count	P_Value	Benjamini
UP_SEQ_FEATURE	DNA-binding region:Homeobox	4	1.20E-01	1.00E+00
INTERPRO	Homeodomain, metazoa	3	1.20E-01	9.90E-01
UP_KEYWORDS	Homeobox	4	2.20E-01	8.20E-01
INTERPRO	Homeodomain	4	2.40E-01	1.00E+00
INTERPRO	Homeobox, conserved site	3	3.60E-01	1.00E+00
SMART	HOX	4	3.60E-01	1.00E+00
INTERPRO	Homeodomain-like	4	3.90E-01	1.00E+00
	Enrichment Score: 0.46	Count	P_Value	Benjamini
UP_KEYWORDS	Transmembrane helix	39	2.70E-01	8.50E-01
UP_KEYWORDS	Transmembrane	39	2.80E-01	8.40E-01
UP_SEQ_FEATURE	transmembrane region	34	4.10E-01	1.00E+00
GOTERM_CC_DIRECT	integral component of membrane	36	4.50E-01	9.70E-01
	Enrichment Score: 0.42	Count	P_Value	Benjamini
GOTERM_BP_DIRECT	small GTPase mediated signal transductio	4	2.40E-01	1.00E+00
INTERPRO	Small GTP-binding protein domain	3	3.00E-01	1.00E+00
GOTERM_MF_DIRECT	GTP binding	4	4.70E-01	1.00E+00
UP_SEQ_FEATURE	nucleotide phosphate-binding region:GTP	3	5.80E-01	1.00E+00
	Enrichment Score: 0.39	Count	P_Value	Benjamini
UP_KEYWORDS	Transcription regulation	17	3.50E-01	8.50E-01
UP_KEYWORDS	Transcription	17	4.00E-01	8.50E-01
GOTERM_BP_DIRECT	transcription, DNA-templated	15	4.90E-01	1.00E+00
	Enrichment Score: 0.31	Count	P_Value	Benjamini
UP_SEQ_FEATURE	zinc finger region:C2H2-type 4	6	3.00E-01	1.00E+00
UP_SEQ_FEATURE	zinc finger region:C2H2-type 7	5	3.30E-01	1.00E+00
UP_SEQ_FEATURE	zinc finger region:C2H2-type 3	6	3.60E-01	1.00E+00
UP_SEQ_FEATURE	zinc finger region:C2H2-type 5	5	4.50E-01	1.00E+00
INTERPRO	Zinc finger, C2H2-like	6	5.70E-01	1.00E+00

INTERPRO	Zinc finger, C2H2	6	6.20E-01	1.00E+00
INTERPRO	Zinc finger C2H2-type/integrase DNA-bir	5	7.00E-01	1.00E+00
SMART	ZnF_C2H2	6	7.90E-01	1.00E+00
	Enrichment Score: 0.29	Count	P_Value	Benjamini
INTERPRO	WD40/YVTN repeat-like-containing dom	4	3.80E-01	1.00E+00
UP_KEYWORDS	WD repeat	3	5.20E-01	9.00E-01
UP_SEQ_FEATURE	repeat:WD 2	3	5.20E-01	1.00E+00
UP_SEQ_FEATURE	repeat:WD 1	3	5.20E-01	1.00E+00
INTERPRO	WD40 repeat	3	5.30E-01	1.00E+00
SMART	WD40	3	6.70E-01	1.00E+00
	Enrichment Score: 0.24	Count	P_Value	Benjamini
INTERPRO	Immunoglobulin subtype	5	4.20E-01	1.00E+00
UP_KEYWORDS	Immunoglobulin domain	5	4.30E-01	8.70E-01
SMART	IG	5	6.20E-01	1.00E+00
INTERPRO	Immunoglobulin-like domain	5	7.60E-01	1.00E+00
INTERPRO	Immunoglobulin-like fold	6	7.60E-01	1.00E+00
	Enrichment Score: 0.23	Count	P_Value	Benjamini
UP_SEQ_FEATURE	zinc finger region:C2H2-type 10	4	3.60E-01	1.00E+00
UP_SEQ_FEATURE	zinc finger region:C2H2-type 9	4	4.30E-01	1.00E+00
UP_SEQ_FEATURE	zinc finger region:C2H2-type 12	3	4.80E-01	1.00E+00
UP_SEQ_FEATURE	zinc finger region:C2H2-type 8	4	5.00E-01	1.00E+00
UP_SEQ_FEATURE	zinc finger region:C2H2-type 11	3	5.60E-01	1.00E+00
UP_SEQ_FEATURE	zinc finger region:C2H2-type 6	4	6.10E-01	1.00E+00
UP_SEQ_FEATURE	domain:KRAB	3	6.30E-01	1.00E+00
INTERPRO	Krueppel-associated box	3	7.50E-01	1.00E+00
SMART	KRAB	3	8.50E-01	1.00E+00
UP_SEQ_FEATURE	zinc finger region:C2H2-type 2	3	9.00E-01	1.00E+00
	Enrichment Score: 0.17	Count	P_Value	Benjamini
UP_SEQ_FEATURE	domain:Protein kinase	4	5.80E-01	1.00E+00
INTERPRO	Serine/threonine-protein kinase, active site	3	6.20E-01	1.00E+00
INTERPRO	Protein kinase, catalytic domain	4	6.30E-01	1.00E+00
UP_SEQ_FEATURE	binding site:ATP	4	6.80E-01	1.00E+00
INTERPRO	Protein kinase-like domain	4	6.90E-01	1.00E+00
GOTERM_MF_DIRECT	protein kinase activity	3	6.90E-01	1.00E+00
UP_KEYWORDS	Serine/threonine-protein kinase	3	7.00E-01	9.60E-01
GOTERM_MF_DIRECT	protein serine/threonine kinase activity	3	7.20E-01	1.00E+00
UP_SEQ_FEATURE	active site:Proton acceptor	4	7.90E-01	1.00E+00
	Enrichment Score: 0.02	Count	P_Value	Benjamini
UP_KEYWORDS	Transducer	4	9.20E-01	1.00E+00
UP_KEYWORDS	G-protein coupled receptor	3	9.70E-01	1.00E+00
GOTERM_BP_DIRECT	G-protein coupled receptor signaling pathway	3	9.90E-01	1.00E+00

Supplementary Table 5 List of siRNA2 target gene annotation by DAVID				
	Enrichment Score: 1.44	Count	P_Value	Benjamini
GOTERM_CC_DIRECT	pre-autophagosomal structure	3	6.80E-03	7.30E-01
GOTERM_BP_DIRECT	autophagosome assembly	3	3.40E-02	9.90E-01
UP_KEYWORDS	Autophagy	3	2.10E-01	8.20E-01
	Enrichment Score: 1.03	Count	P_Value	Benjamini
GOTERM_BP_DIRECT	mRNA 3'-end processing	3	4.80E-02	9.70E-01
GOTERM_BP_DIRECT	termination of RNA polymerase II transcr	3	7.40E-02	9.90E-01
KEGG_PATHWAY	mRNA surveillance pathway	3	1.10E-01	1.00E+00
GOTERM_BP_DIRECT	mRNA splicing, via spliceosome	4	2.10E-01	1.00E+00
	Enrichment Score: 0.98	Count	P_Value	Benjamini
SMART	PDZ	4	8.00E-02	1.00E+00
INTERPRO	PDZ domain	4	9.00E-02	1.00E+00
UP_SEQ_FEATURE	domain:PDZ	3	1.60E-01	1.00E+00
	Enrichment Score: 0.95	Count	P_Value	Benjamini
UP_KEYWORDS	Translation regulation	4	3.10E-02	5.10E-01
UP_KEYWORDS	mRNA transport	4	3.10E-02	4.50E-01
GOTERM_BP_DIRECT	RNA splicing	3	3.30E-01	1.00E+00
UP_KEYWORDS	mRNA splicing	3	5.00E-01	9.30E-01
	Enrichment Score: 0.93	Count	P_Value	Benjamini
UP_SEQ_FEATURE	short sequence motif:Selectivity filter	3	5.20E-02	1.00E+00
GOTERM_BP_DIRECT	potassium ion transport	3	1.10E-01	9.90E-01
GOTERM_CC_DIRECT	voltage-gated potassium channel complex	3	1.10E-01	9.60E-01
UP_KEYWORDS	Potassium transport	3	1.70E-01	8.70E-01
UP_KEYWORDS	Potassium	3	2.00E-01	8.30E-01
	Enrichment Score: 0.56	Count	P_Value	Benjamini
UP_KEYWORDS	Transmembrane helix	42	2.10E-01	8.20E-01
UP_KEYWORDS	Transmembrane	42	2.20E-01	8.20E-01
UP_SEQ_FEATURE	transmembrane region	37	2.70E-01	1.00E+00
GOTERM_CC_DIRECT	integral component of membrane	36	4.50E-01	9.90E-01
	Enrichment Score: 0.51	Count	P_Value	Benjamini
SMART	ANK	4	2.40E-01	1.00E+00
UP_KEYWORDS	ANK repeat	4	2.40E-01	8.40E-01
INTERPRO	Ankyrin repeat	4	2.50E-01	1.00E+00
INTERPRO	Ankyrin repeat-containing domain	4	2.70E-01	1.00E+00
UP_SEQ_FEATURE	repeat:ANK 1	3	4.70E-01	1.00E+00
UP_SEQ_FEATURE	repeat:ANK 2	3	4.70E-01	1.00E+00
	Enrichment Score: 0.47	Count	P_Value	Benjamini
GOTERM_BP_DIRECT	transcription, DNA-templated	17	3.00E-01	1.00E+00
UP_KEYWORDS	Transcription regulation	18	3.40E-01	8.60E-01
UP_KEYWORDS	Transcription	18	3.80E-01	8.80E-01
	Enrichment Score: 0.43	Count	P_Value	Benjamini
SMART	PH	4	2.60E-01	1.00E+00
INTERPRO	Pleckstrin homology domain	4	2.80E-01	1.00E+00
UP_SEQ_FEATURE	domain:PH	3	4.70E-01	1.00E+00
INTERPRO	Pleckstrin homology-like domain	4	5.60E-01	1.00E+00
	Enrichment Score: 0.42	Count	P_Value	Benjamini
UP_SEQ_FEATURE	domain:Fibronectin type-III 4	3	6.20E-02	1.00E+00
UP_SEQ_FEATURE	domain:Fibronectin type-III 3	3	1.00E-01	1.00E+00
UP_SEQ_FEATURE	domain:Fibronectin type-III 2	3	2.10E-01	1.00E+00
UP_SEQ_FEATURE	domain:Fibronectin type-III 1	3	2.10E-01	1.00E+00
INTERPRO	Immunoglobulin I-set	3	2.50E-01	1.00E+00
SMART	FN3	3	2.70E-01	1.00E+00
INTERPRO	Fibronectin, type III	3	4.20E-01	1.00E+00

SMART	IGc2	3	5.10E-01	1.00E+00
INTERPRO	Immunoglobulin subtype 2	3	5.10E-01	1.00E+00
SMART	IG	3	8.60E-01	1.00E+00
INTERPRO	Immunoglobulin subtype	3	8.60E-01	1.00E+00
UP_KEYWORDS	Immunoglobulin domain	3	8.70E-01	1.00E+00
INTERPRO	Immunoglobulin-like fold	4	9.60E-01	1.00E+00
INTERPRO	Immunoglobulin-like domain	3	9.70E-01	1.00E+00
	Enrichment Score: 0.36	Count	P_Value	Benjamini
INTERPRO	EF-hand domain	4	2.10E-01	1.00E+00
UP_SEQ_FEATURE	domain:EF-hand 2	3	3.30E-01	1.00E+00
UP_SEQ_FEATURE	domain:EF-hand 1	3	3.30E-01	1.00E+00
INTERPRO	EF-Hand 1, calcium-binding site	3	3.40E-01	1.00E+00
INTERPRO	EF-hand-like domain	3	5.70E-01	1.00E+00
GOTERM_MF_DIRECT	calcium ion binding	5	7.10E-01	1.00E+00
UP_KEYWORDS	Calcium	4	9.30E-01	1.00E+00
	Enrichment Score: 0.17	Count	P_Value	Benjamini
UP_SEQ_FEATURE	zinc finger region:C2H2-type 2	5	5.60E-01	1.00E+00
UP_SEQ_FEATURE	zinc finger region:C2H2-type 3	5	5.90E-01	1.00E+00
INTERPRO	Zinc finger, C2H2	6	6.40E-01	1.00E+00
INTERPRO	Zinc finger C2H2-type/integrase DNA-bir	5	7.20E-01	1.00E+00
SMART	ZnF_C2H2	5	7.70E-01	1.00E+00
INTERPRO	Zinc finger, C2H2-like	5	7.70E-01	1.00E+00
	Enrichment Score: 0.1	Count	P_Value	Benjamini
UP_SEQ_FEATURE	zinc finger region:C2H2-type 9	3	7.10E-01	1.00E+00
UP_SEQ_FEATURE	zinc finger region:C2H2-type 8	3	7.60E-01	1.00E+00
UP_SEQ_FEATURE	zinc finger region:C2H2-type 7	3	8.00E-01	1.00E+00
UP_SEQ_FEATURE	zinc finger region:C2H2-type 6	3	8.40E-01	1.00E+00
UP_SEQ_FEATURE	zinc finger region:C2H2-type 4	3	9.00E-01	1.00E+00
	Enrichment Score: 0.09	Count	P_Value	Benjamini
UP_SEQ_FEATURE	nucleotide phosphate-binding region:ATP	6	7.70E-01	1.00E+00
UP_KEYWORDS	ATP-binding	8	8.00E-01	9.90E-01
GOTERM_MF_DIRECT	ATP binding	8	8.80E-01	1.00E+00
